# Healthcare professionals' satisfaction toward the use of district health information system and its associated factors in southwest Ethiopia: using the information system success model

**DOI:** 10.3389/fdgth.2023.1140933

**Published:** 2023-07-17

**Authors:** Agmasie Damtew Walle, Addisalem Workie Demsash, Tigist Andargie Ferede, Sisay Maru Wubante

**Affiliations:** ^1^ Department of Health Informatics, College of Health Science, Mettu University, Mettu, Ethiopia; ^2^ Department of Epidemiology and Biostatistics, Institute of Public Health, College of Medicine and Health Sciences, University of Gondar, Gondar, Ethiopia; ^3^ Department of Health Informatics, Institute of Public Health, College of Medicine and Health Sciences, University of Gondar, Gondar, Ethiopia

**Keywords:** DHIS2, satisfaction, healthcare professionals, Ethiopia, D&M model

## Abstract

**Background:**

Ethiopia has the potential to use the district health information system, which is a building block of the health system. Thus, it needs to assess the performance level of the system by identifying the satisfaction of end users. There is little evidence about users' satisfaction with using this system. As a result, this study was conducted to fill this gap by evaluating user satisfaction and associated factors of district health information system among healthcare providers in Ethiopia, using the information system success model.

**Methods:**

An institutional-based cross-sectional study was conducted from November to December 2022 in the Oromia region of southwest Ethiopia. A total of 391 health professionals participated in the study. The study participants were selected using a census. Using a self-administered questionnaire, data were collected. Measurement and structural equation modeling analyses were used to evaluate reliability, the validity of model fit, and to test the relationship between the constructs, respectively, using analysis of moment structure (AMOS) V 26.

**Results:**

System quality had a positive direct effect on the respondent's system use (*β* = 0.18, *P*-value < 0.001), and satisfaction (*β* = 0.44, *P*-value < 0.001). Service quality had also a direct effect on the respondent's system use (*β* = 0.37, *P*-value < 0.01), and satisfaction with using the district health information system (*β* = 0.36, *P*-value < 0.01). Similarly, system use had also a direct effect on the respondent's satisfaction (*β* = 0.53, *P*-value < 0.05). Moreover, computer literacy had a direct effect on the respondent's system use (*β* = 0.63, *P*-value < 0.05), and satisfaction (*β* = 0.51, *P*-value < 0.01).

**Concussions:**

The overall user satisfaction with using the district health information system in Ethiopia was low. System quality, service quality, and computer literacy had a direct positive effect on system use and user satisfaction. In addition, system use and information quality had a direct positive effect on healthcare professionals' satisfaction with using the district health information system. The most important factor for enhancing system use and user satisfaction was computer literacy. Accordingly, for the specific user training required for the success of the district health information system in Ethiopia, the manager should offer additional basic computer courses for better use of the system.

## Background

The formulation of decisions, policies, and plans is based on the use of exact, valid, timely, and reliable data and information (1, 2). Achieving better health outcomes requires robust health systems, and moving forward, a strong health system is built on a strong health information system (HIS) (3). Globally, to achieve universal health coverage, information systems are crucial and are key in health interventions, assessments of the health sector, planning, resource allocation, and program oversight (4). The World Health Organization states that the main goal of HIS is the global development of automated patient data services, which results in more effectively retrieving the data needed for treatment, statistics, teaching, and research. To improve the efficacy and effectiveness of health care through better management at all levels, HISs are developed for the integrated collection of data, processing, and reporting (5).

District Health Information System Version 2 (DHIS2) is a web-based, integrated national health information system that incorporates high-quality data used at all levels to enhance the delivery of healthcare services (3, 6, 7). More than 60 countries currently use DHIS, and most international projects are more interested in using it to track health performance (7, 8). Ethiopia has also developed DHIS usage potentials that will roll out user-friendly DHIS 2 versions throughout the region. To improve decision-making among public health facilities, the Federal Ministry of Health (FMOH) is deploying and implementing DHIS 2.

Information systems (IS) demonstrate a relationship between users' attitudes and intentions to continue with information systems. As a result, the increase in IS investments highlights the need for understanding end-user satisfaction and system usage. Additionally, it is claimed that user satisfaction, a subjective or perceptual metric associated with users' attitudes and keeping intentions, has been widely used to assess the success of IS (9). One of the most common methods for assessing a system's performance and, consequently, its success is user satisfaction (5, 10). User satisfaction, satisfaction with the hardware and software, satisfaction with the system development project, user complaints about the information system center, and user satisfaction with the intermediaries are factors relating to system success (11, 12).

According to an Iranian study, satisfaction criteria in 11 different hospitals were relatively favorable (54.6%) (10). Another study conducted in Tanzania showed that 85% of the users responded that they were satisfied with the DHIS2 system (9). A study done about the utilization of DHIS-2 among healthcare professionals in southwest Ethiopia was 57.3% and the study participants who were serving in the expert position had more good utilization of DHIS-2 (49.1%) (7). But the satisfaction of those users with this system was uncertain. One classifies a system as weak if it does not meet the needs of the users and is not consumer-based (13). Disregarding or not paying enough attention to human aspects is one of the primary reasons why an information system fails to accomplish some of its intended aims. This, in turn, would fail to create an appropriate interface between the system and its users and to provide the users with a sense of ownership of the system (12).

Organizations have used benchmarking, Kaplan and Norton's balanced scorecard, and research has created models like DeLone and McLean's (D&M), and Doll's to better understand the tangible and intangible benefits of IS implementation, highlighting the need for better and more reliable success metrics (9, 14). Although the majority of health professionals believe that technology may reduce the burden of paper-based documentation and the inaccessibility of patient data in urgent situations, they can also be quickly dissatisfied when a new system or support does not meet their expectations (15, 16).

### Theoretical background and hypothesis

**Figure 1:**
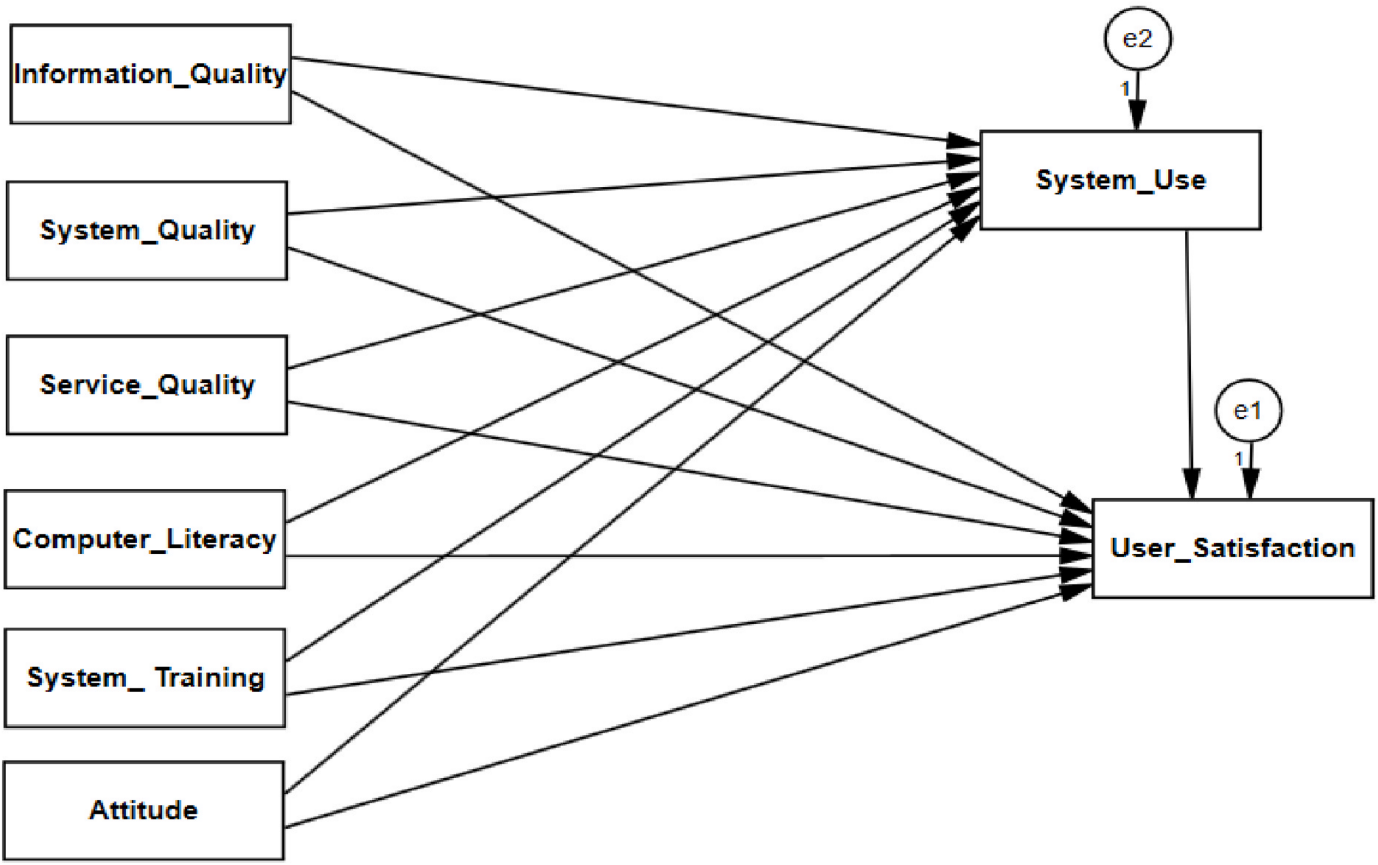
Modified information system success model.

DeLone and Mclean models offer a model that encompasses the dimensions for IS success measurement after thoroughly reviewing various IS success measurements (17).In this study, we proposed a modified DeLone and McLean's (D&M) model that consisted of five main components (information quality, system quality, service quality, system use, and user satisfaction) with the addition of three new factors (computer literacy, system training, and attitude), which were significant factors for users' system use and satisfaction with DHIS2 (10–12, 15, 16), and the following provides details of adapting predictors and our hypotheses (Figure 1).

### System quality

System quality examines whether a system has the user-required capability to support the activity at work, and researchers have found that the most popular indicator of system quality is the ease of use (18). System quality identified as the primary determinant factor for IS success measurement proposed by various studies (17–19). In the context of this, the following hypotheses are investigated in this study:

H1: System quality will have a positive effect on DHIS-2 use.
H2: System quality will have a positive effect on user satisfaction

### Information quality

Information quality concerns are associated with IS output metrics. The majority of proven information quality measures include perceived usefulness, accuracy, format, and timeliness (17). Information quality is identified as the determinant factor for IS use and user satisfaction (17, 18, 20). Accordingly, the following hypotheses are investigated in this study:

H3: Information quality will have a positive effect on DHIS-2 use.
H4: Information quality will have a positive effect on user satisfaction

### Service quality

Service quality takes into account the external and internal user support that is offered, as well as the extra infrastructures that support the proper adoption of the DHIS-2 (18). A study was done in America (21), Brazil (22), Denmark (23), Nigeria (24), and Ethiopia (15). showed that, service quality was identified as the factor to influence IS use and user satisfaction. Thus, this study tests the following hypotheses:

H5: Service quality will have a positive effect on DHIS-2 use.
H6: Service quality will have a positive effect on user satisfaction

### Computer literacy

Computer literacy is the term used to describe the knowledge and abilities that allow individuals to use computers efficiently for a certain purpose (18). Studies conducted in UAE (25), Saudi Arabia (26), and Ethiopia (18).revealed that commuter literacy was a determinant factor for IS success. As a result, this study tests the following hypotheses:

H7: Computer literacy will have a positive effect on DHIS-2 use.
H8: Computer literacy will have a positive effect on user satisfaction

### Attitude

Attitude exhibits how individuals' thoughts toward the information system affect their feelings and behavior, and the study revealed that attitude/feeling towards the system influences the information system (16). This study tests the following hypotheses:

H9: Attitude will have a positive effect on DHIS-2 use.
H10: Attitude will have a positive effect on user satisfaction

### System training

System training specifically the training initiatives that are designed to teach the use of health information systems (HMIS, EMRs, DHIS-2) to the users (27). studies revealed that system use influences IS success (15, 27). Hence, this study tests the following hypothesis:

H11: System training will have a positive effect on DHIS-2 use.
H12: System training will have a positive effect on user satisfaction

### System use

System use is the actual use of the district health information system, and studies revealed that system use influences user satisfaction (28, 29). In the context of this, the study tests the following hypotheses:

H13: System use will have a positive effect on user satisfaction
H14: System use mediates the relationship between information quality and DHIS-2 user satisfaction
H15: System use mediates the relationship between system quality and DHIS-2 user satisfaction
H16: System use mediates the relationship between service quality and DHIS-2 user satisfaction
H17: System use mediates the relationship between user attitude and DHIS-2 user satisfaction
H18: System use mediates the relationship between computer literacy and DHIS-2 user satisfaction
H19: System use mediates the relationship between system training and DHIS-2 user satisfaction

The determining elements that contributed to the success of the information system in those settings can differ from those in developed nations. Therefore, to understand the crucial success and failure criteria, rigorous assessment studies on various health information system implementation projects in such settings are required.

The study may have effects on practice, policy, and upcoming researchers. The beneficiaries of this study are health professionals, healthcare organizations. and patients. Ethiopia has the potential to use the district health information system, which is a building blocks of the health system. Thus, it needs to assess the performance level of the system by identifying the satisfaction of end users. According to our search of the literature, little research has been done on the subject of healthcare professionals' satisfaction with using district health information systems in a resource-limited setting using the information system success model. As a result, the purpose of and specific alim of this study was conducted to Introduce a modified theoretical model constructed based on the on the information technology success model (D&M model) and empirically test the modified information system success model for determining the key factors influencing satisfaction of healthcare providers towards using DHIS2, which was implemented in southwest Ethiopia.

## Methods

### Study setting and period

This study was carried out in public facilities in Ilu Abba Bor and Buno Bedelle Zones, Oromia Regional State, southwest Ethiopia. Ilu Abba Bor Zone and Buno Bedelle Zone are two of the 20 zones of the Oromia regional state situated southwest of the region and located at a distance of about 600 km and 483 km from the center of the region, respectively. In the two zones, there are six public hospitals, namely: Bedele Hospital, Darimu Hospital, Dembi Hospital, Metu Karl Hospital, Dedhesa Hospital, and Chora Hospital. The study was conducted from November to December 2022.

### Study design

An institution-based cross-sectional study was carried out among healthcare professionals who were working in public hospitals.

### The population of the study

All health professionals who were working in Ilu AbaBor and Bunno Bedelle zone public hospitals were considered the source population, whereas all health professionals who were working in the system at Ilu AbaBor and Bunno Bedelle zone public hospitals during the study period were considered the study population. However, healthcare professionals, who worked in the system and were not available during the study period, and had less than six months of work experience not included in the study.

### Sample size determination and sampling procedure

Study participants were included from six hospitals in southwest Ethiopia. The data was collected by approaching each study participant. Selected hospitals found within the research areas were contacted. Based on study participants at each hospital, the sample size was calculated. Hence, all health professionals who handle data, generate data, use generated data for their decision-making, and serve as the focal person within their hospitals were included, and the users of DHIS-2 in southwest Ethiopia were a small number (*n* = 421). Thus, the study participants were sampled using census in selected hospitals.

### Operational definitions

**Health professionals:** Users who serve as the focal person within their department.to handle data, generate data and use generated data for their decision-making, which included physicians (doctors and health officers), nurses (clinical nurses, midwives, optometrists, physiotherapists, and anesthesiologists), laboratories, pharmacies, radiologists, and HMIS (health data entry and management secretaries, and information system officers) (15, 30).

**User satisfaction**: Satisfaction was assessed using a 5-point Likert scale with a 5-item questionnaire, participants with a score equal to or above the median were categorized as satisfied; those with scores below the median were categorized as dissatisfied (15, 31).

### Data collection tool, data quality, and procedures

A self-administered questionnaire was developed based on standardized and previously validated tools (Supplementary File). The questions were categorized into two sections. The first section focuses on the user's sociodemographic characteristics with five questions. The second section about the information system success evaluation model variables, which was developed by DeLone and MacLean (D&M), was used as the basis for this study (15, 17). In the world of informatics, a validated and widely used information system success evaluation approach (15). System quality; 7 items, information quality; 10 items, service quality; 9 items, system use; 10 items, user satisfaction; 5 items, and net benefit are the fundamental dimensions in this paradigm (7, 9–12, 17, 32, 33). To evaluate user satisfaction, we selected five parameters from the D&M model while omitting the net benefit. User background factors like system training; 4 items, and computer literacy; 4 items were included as determining factors to be examined instead of net benefit because numerous researchers identified it as a determinant element, particularly in low resource settings (15, 33). In addition, we extend the attitude; items that influence the user satisfaction towards using DHIS2 (9, 34).

A pretest study with 10% of the total sample size was undertaken out of the study in Jimma Hospital before the actual data collection to assess the validity and reliability of the data collection instrument. The required adjustments were therefore made.

The internal consistency of each component of the data collection tool was assessed using Cronbach's alpha, which was obtained from system quality = 0.83, information quality = 0.81, service quality = 0.79, system use = 0.91, user satisfaction = 0.88, attitudes = 0.84, system training = 0.85, computer literacy = 0.81. Finally, two days of training for the actual data collection were given to three onsite supervisors, three health informatics, and two nurse professionals who served as data collectors. With eligible study participants, the validated data-collecting instrument was used to gather data, and the consistency and completeness of the data were also reviewed daily by the supervisors and investigators.

### Data processing and analysis

Data were entered using Epi Data version 4.0.2, and descriptive analysis was done using STATA version 14. For descriptive statistics, frequencies and percentages were determined and presented using graphs and tables, Moreover, model predictors were analyzed using structural equation model (SEM) software called analysis of moment structure (AMOS) version 26. Standardized path coefficients were used to identify the association between predictors and dependent variables.

This study also used the common model-fit measures to assess the model's overall goodness of fit, including the Chi-square ratio (<3), the goodness of fit index (GFI > 0.9), adjusted goodness of fit index (AGFI > 0.8), normal fit index (NFI > 0.9) and root mean square of standardized residual (RMSR <0 .08) to evaluate overall model fitness. The result demonstrates that the measurement model exhibited a fairly good fit with the data collected (*χ*^2^/d.f. = 2.25, GFI = 0.94, AGFI = 0.90, NFI = 0.94, RMSR = 0.06) (17, 35, 36).

To determine statistically significant predictors the critical ratio and standardized path coefficients with *P*-value <0.05 were employed to assess the relationship between the predictors and dependent variable. The influence and level of significance of each of the six possible mediation paths in the model were explored, partial mediation occurs when a construct's direct, indirect, and total effects are all statistically significant, and full mediation occurs when the direct and indirect effects are both statistically significant but the total effect is not. Moreover, to confirm a mediation effect, we typically looked for a substantial indirect effect with a *P*-value < 0.05.

## Results

### Sociodemographic characteristics

Out of 421 participants in this study, 391 (92.87% response rate) of them completed the questionnaire. Of all participants, 218 (55.8%) were male. Around three-fifth, 231 (59.1%) of the participant's age ranged from 20 to 30 years with the mean age of the participant was 31 years ± 5.9 SD. More than half of 217 (55.5%) of the participants ranges between 3 and 5 years of work experience. Around three-fifth 250 (63.9%) of the participants had a salary of between 5,000–10,000 ETB (Table 1).

**Table 1:** Sociodemographic characteristics of healthcare professionals in southwest Ethiopia, 2022 (*n* = 391).

Variable	Category	Frequency	Percent
Gender	Female	173	44.2
	Male	218	55.8
Age (year)	20–30	231	59.1
	31–40	132	33.8
	>40	28	7.1
Experience (year)	<3	60	15.3
	3–5	217	55.5
	>5	114	29.2
Salary (ETB)	<5,000	108	27.6
	5,000–10,000	250	63.9
	>10,000	33	8.5

### User satisfaction level of DHIS2

**Figure 2:**
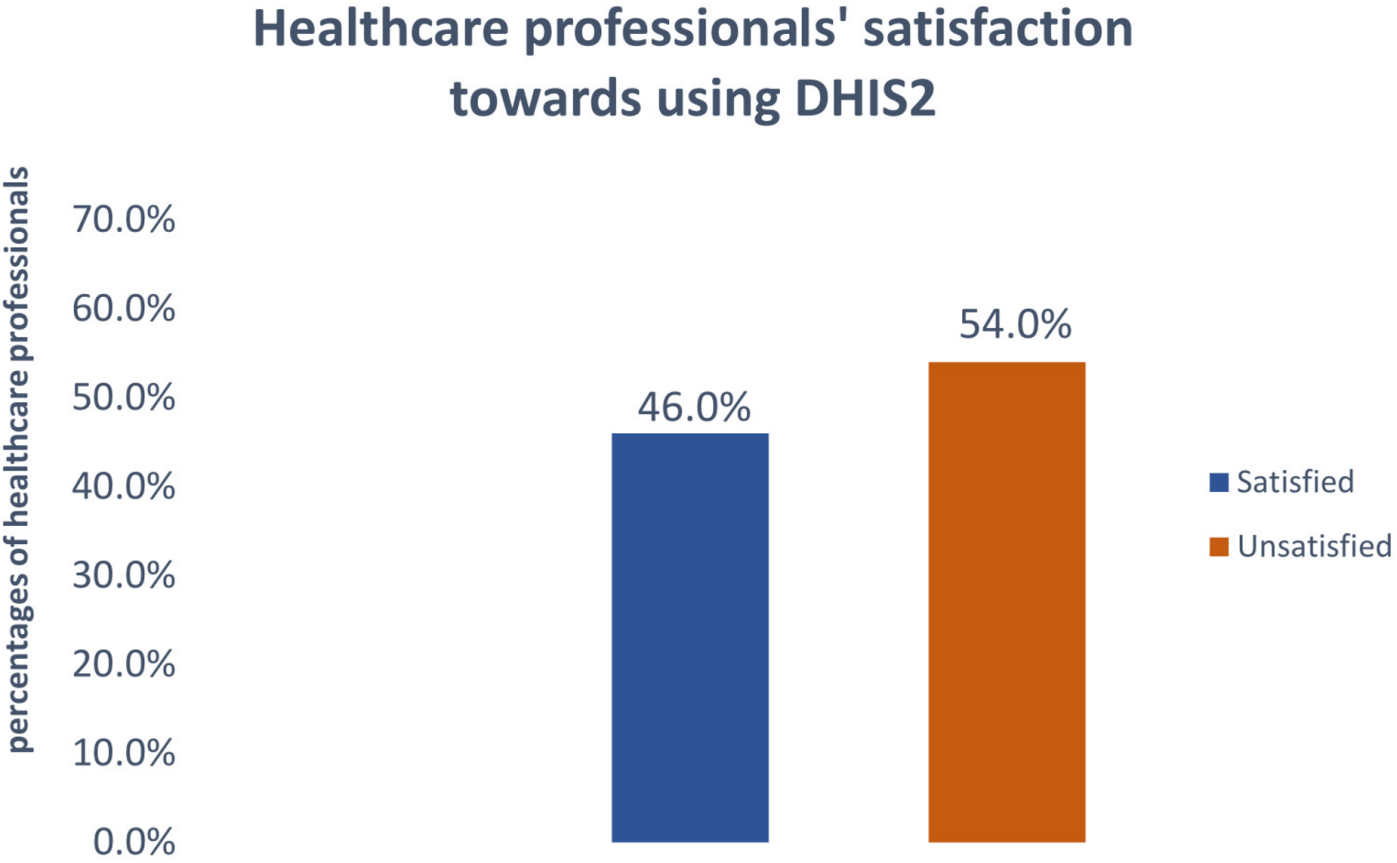
Proportion of satisfaction towards using DHIS2 among healthcare professionals, southwest Ethiopia, 2022.

In this study, 180 (46.0%) (95.0%: CI:41.2–50.9) study participants, were satisfied with district health information (DHIS2) (Figure 2). The median score of satisfaction with using district health information (DHIS2) was 15 (IQR = 11–20), and the minimum and maximum scores were 5 and 25, respectively.

### Factors associated with DHIS2 user satisfaction

**Figure 3:**
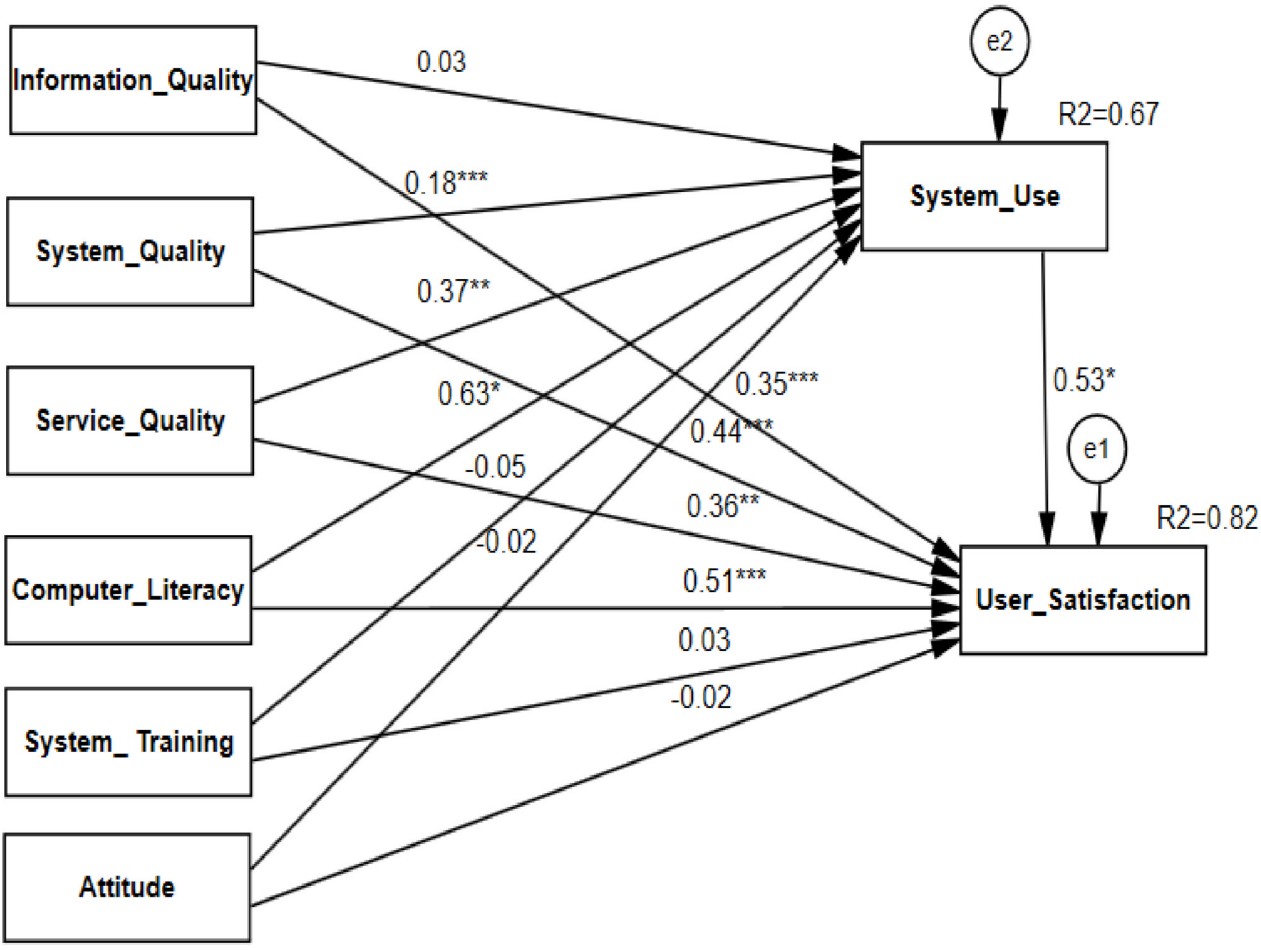
SEM analysis of associated factors of healthcare professionals’ satisfaction towards using DHIS2, southwest Ethiopia, 2022.

SEM analysis found that information quality, system quality, service quality, computer literacy, system training, and attitude was explained in 67% of system use and 82.0% of the endogenous variable (DHIS2 user satisfaction), which had an R^2^ of 0.67 and 0.82 respectively. This showed that the proposed model was a strong predictive power. Further, the results offer significant insights into healthcare providers' satisfaction with using DHIS2 for improving the quality of the healthcare system in a resource-limited setting. The model's standardized estimates of the model predictors are shown below (Figure 3).

The SEM analysis finding presented in Figure 3 showed that system quality had a positive direct effect on the respondent's system use (*β* = 0.18, *P*-value < 0.001), and satisfaction towards using DHIS2 (*β* = 0.44, *P*-value < 0.001). This shows that one standard deviation additional change in system quality increases the use of the system by 0.18 units and the satisfaction level of healthcare professionals towards using DHIS2 by 0.44 units standard deviation keeping another variable constant. Service quality had also a positive direct effect on the respondent's system use (*β* = 0.37, *P*-value < 0.01), and satisfaction with using DHIS2 (*β* = 0.36, *P*-value < 0.01). This shows that one standard deviation additional change in service quality increases the use of the system by 0.37 units and the satisfaction level of healthcare professionals towards using DHIS2 by 0.36-unit standard deviation keeping another variable constant.

Similarly, system use had also a positive direct effect on the respondent's satisfaction with using DHIS2 (*β* = 0.53, *P*-value < 0.05). This shows that with one standard deviation increases in system use the satisfaction level of healthcare professionals towards using DHIS2 raised by 0.53-unit standard deviation keeping another variable constant. Moreover, the finding showed that computer literacy had a positive direct effect on the respondent's system use (*β* = 0.63, *P*-value < 0.05), and satisfaction with using DHIS2 (*β* = 0.51, *P*-value < 0.01). This shows that one standard deviation additional change in computer literacy increases the use of the system by 0.63 units and the satisfaction level of healthcare professionals towards using DHIS2 by 0.51-unit standard deviation keeping another variable constant.

However, the study revealed that system training had not a positive direct effect on the respondent's system use (*β* = −0.05, *P*-value = 0.316), and satisfaction with using DHIS2 (*β* = 0.03, *P*-value = 0.576). and attitude had also not a positive direct effect on the respondent's system use (*β* = −0.02, *P*-value = 0.620), and satisfaction towards using DHIS2 (*β* = −0.02, *P*-value = 0.690). In addition, information quality was not having a significant effect on system use (*β* = 0.03, *P*-value = 0.569) among healthcare professionals in Ethiopia.

In summary, system use had the most substantial effect on the respondent's satisfaction towards using DHIS2, and computer literacy had the most important factor towards using DHIS2, which was larger than the effects of other predictors (Table 2).

**Table 2:** SEM analysis of healthcare professionals’ satisfaction towards using district health information system and its associated factors in southwest Ethiopia, 2022.

Path	Hn	Estimate	S.E.	C.R.	*P*-Value	Decision
System Use <— System Quality	H1	0.181	0.053	3.433	***	Supported
User Satisfaction <— System Quality	H2	0.441	0.051	4.225	***	Supported
System Use <— Information Quality	H3	0.029	0.050	0.570	0.569	Not supported
User Satisfaction <— Information Quality	H4	0.353	0.048	7.761	***	Supported
System Use <— Service Quality	H5	0.370	0.046	2.621	0.009**	Supported
User Satisfaction <— Service Quality	H6	0.362	0.044	2.859	0.004**	Supported
System Use <— Computer_Literacy	H7	0.629	0.047	2.516	0.012*	Supported
User Satisfaction <—Computer_Literacy	H8	0.513	0.046	4.627	***	Supported
System Use <— Attitude	H9	−0.022	0.044	−0.495	0.620	Not supported
User Satisfaction <— Attitude	H10	−0.017	0.042	−0.399	0.690	Not supported
System Use <— System Training	H11	−0.054	0.084	−1.002	0.316	Not supported
User Satisfaction <— System Training	H12	0.034	0.081	0.559	0.576	Not supported
User Satisfaction <— System Use	H13	0.527	0.049	2.557	0.015*	Supported

*** significant at *P* value < 0.001.

** significant at *P* value < 0.01.

* significant at *P* value < 0.05, Hn: Hypothesis.

### Mediation effect

In this study, the mediation analysis revealed that system use partially mediates the relationship between system quality and user satisfaction with DHIS2 (*β* = 0.304, *P*-value = 0.035). This means system use would have an indirect but significant effect on the relationship between system quality and DHIS2 user satisfaction. Furthermore, system use fully mediates the relationship between computer literacy and DHIS2 user satisfaction (*β* = 0.413, *P*-value = 0.011). This means system use would have an indirect but significant effect on the relationship between computer literacy and DHIS2 user satisfaction. However, the system used did not mediate the relationship between information quality, service quality, attitude, and system training with DHIS2 user satisfaction in Ethiopia (Table 3).

**Table 3:** Mediation analysis of healthcare professionals’ satisfaction towards using district health information system and its associated factors in southwest Ethiopia, 2022.

Path	Hn	Effect	Estimate	*P*-value	Result
Information Quality → System Use → User Satisfaction	H14	Total effect	0.347	0.001**	Direct relationship
		Indirect effect	−0.001	0.500	
		Direct effect	0.348	0.003**	
System Quality → System Use → User Satisfaction	H15	Total effect	0.196	0.002**	Partial mediation
		Indirect effect	0.304	0.035*	
		Direct effect	0.192	0.002**	
Service Quality → System Use → User Satisfaction	H16	Total effect	0.126	0.020*	Direct relationship
		Indirect effect	−0.003	0.419	
		Direct effect	0.129	0.019*	
Attitude → System Use → User Satisfaction	H17	Total effect	−0.017	0.680	No relationship
		Indirect effect	0.001	0.490	
		Direct effect	−0.018	0.674	
Computer Literacy → System Use → User Satisfaction	H18	Total effect	0.206	0.003**	Full mediation
		Indirect effect	0.413	0.011*	
		Direct effect	0.209	0.102	
System Training → System Use → User Satisfaction	H19	Total effect	0.026	0.563	No relationship
		Indirect effect	0.001	0.356	
		Direct effect	0.025	0.581	

** Significant at *P* value < 0.01.

* Significant at *P* value < 0.05.

## Discussion

The purpose of the study was to assess healthcare professional satisfaction and its determinant factors towards using DHIS2 among healthcare professionals in southwest Ethiopia. Accordingly, the overall user satisfaction with using DHIS2 was 46.0% [95.0%; CI: (41.2–50.9)]. This finding was consistent with the study done in Malaysia (37). Oman (38), Saudi Arabia (39), and Kenya (40).However, this study's finding was lower than the study conducted in Tanzania (85%) (9). This difference may be due to the system allowing them to locate all required registers, track patients, make transfers and referrals, and receive notifications of lab results. It was simple to learn, understand, and verify data entry and reports, and it was web-based and practical for day-to-day work. The system's training was also good in Tanzania (9). But our study found higher than central Ethiopia user satisfaction with using EMR systems(38.6%) (15). The discrepancy might be due to the system compatibility, user-friendly or easiness between systems. In addition, the study period and sample size between them were also different.

The finding is also lower than a pilot study on district health information system challenges and lessons learned in central Ethiopia (48.65) (7). The possible reason was the study period, sample size, and challenges in southwest Ethiopia such as, high human resource turnover, inadequate access to DHIS skilled personnel, inadequate knowledge of health information (1).

The SEM result showed that system quality, service quality, and computer literacy was a direct positive effect on system use and user satisfaction. In addition, system use and information quality had also a direct positive effect on healthcare professionals' satisfaction with using DHIS2. Hence, H1, H2, H4, H5, H6, H7, H8, H13, H15 and H18 were supported in this study.

System quality had a significant direct and indirect effect on the use of district health information systems and healthcare professionals' satisfaction with using this system. This suggests that increased system quality should result in greater user satisfaction and beneficial effects on personal productivity. This finding is in line with studies about the effect of software quality in Greece (41), system use and user satisfaction in the adoption of electronic medical records systems in Tanzania (9), information systems success in South Africa (17), and electronic medical record system use and user satisfaction at five low-resource setting hospitals in Ethiopia (15). This indicates that users were impressed by the system's various features, such as the ability to access information from any location, follow up on clients, communicate with one another within the system, receive notifications, and store all the registers and client data they need in a centralized location.

Service quality was a direct significant effect on the use of the district health information system and healthcare professionals' satisfaction with using this system. Users will be happier and more likely to use the system if they are more satisfied with the DHIS2 level of support, such as when they receive helpful internal and external assistance. This finding was in line with the study done on user satisfaction with a clinical information system.in America (21), hospital information system satisfaction in Brazil (22), EHR based on the DeLone and McLean model for IS success in Denmark (23), validation of the DeLone and McLean information systems success model in Nigeria (24), and electronic medical record system use and user satisfaction at five low-resource setting hospitals in Ethiopia (15). Accordingly, the IT department's collaboration with the system suppliers in delivering prompt upgrades to the DHIS2 may have increased customer satisfaction with service quality. So, more computers must be placed within the wards so that clinicians may enter patient data without having to wait for a free computer. Improving service quality also requires ensuring a reliable power supply and providing quick system assistance. Given that donor funding supports the majority of installations in such contexts, it is critical for these organizations to offer enough support to improve service quality and, in turn, user satisfaction and DHIS2 use.

Computer literacy was a direct and indirect significant effect on the use of the district health information systems and healthcare professionals' satisfaction with using this system. This is a clear indication that health workers need to have a basic understanding of computers to be more motivated to use the district health information system. This finding was consistent with a study conducted on physician user satisfaction with an electronic medical records system in primary healthcare centers in UAE (25), the association between computer literacy and training on clinical productivity and user satisfaction in using the electronic medical record in Saudi Arabia (26), and modeling antecedents of electronic medical record system implementation success in low-resource setting hospitals in Ethiopia (18). This showed that to increase the success of the district health information system in Ethiopia, it is advised to provide additional basic computer courses during or before system implementation in addition to specific user training (18).

Information quality had a direct positive effect on healthcare professionals “satisfaction with using DHIS2. This demonstrates that higher information quality results in higher user satisfaction and district health information system utilization. The result was consistent with the study done a systematic review of comparison of user groups” perspectives of barriers and facilitators to implementing electronic health records in Canada (20), hospital information system satisfaction in Brazil (22), assessing eGovernment systems success in China (29), and modeling antecedents of electronic medical record system implementation success in low-resource setting hospitals in Ethiopia (18). This revealed that decision-makers should therefore emphasize the following factors when implementing the district health information system: making enough information available, ensuring good accuracy and timely updating of information on the system, and ensuring that reports are in a format and layout that health professionals regularly use and understand (17, 18).

System use also had a direct positive effect on healthcare professionals' satisfaction with using DHIS2. This indicates that users will continue to use the system, thereby increasing their satisfaction with it. This result was consistent with a study done on system use and user satisfaction in the adoption of electronic medical records systems in Tanzania (9) and the measurement and dimensionality of the mobile learning system's success in Taiwan (42). The possible solution might be due to, users believing that since all registers were contained inside one system, they would be able to track their customers within the system with the aid of message notifications in the event of transfers and they would also be able to readily identify the non-valuated clients. Additionally, they believed that with the notification capabilities, regional hospitals could easily and quickly provide them with testing results, as opposed to the past, when they had to wait for a phone call or the post office (9).

### Implications of the study

Based on the results, this study provides theoretical and practical implications to facilitate the successful implementation of DHIS2 in Ethiopia. Theoretically, this study evaluated the effectiveness of DHIS2 in limited resource environments; it is the first full validation of the D&M model in Ethiopia and will provide awareness for system users about how to effectively use the system and increase their satisfaction. In addition, the study will help as a baseline for upcoming research and insight for policymakers to improve the success of the district health information system in Ethiopia. Practically, the managers should focus on significant factors such as system quality, service quality, information quality, and computer literacy for ensuring system use and user satisfaction with using DHIS2 in Ethiopia. Computer literacy was the more powerful and significant factor in improving the system used as well as healthcare professionals' satisfaction with using DHIS2. Accordingly, the manager should provide additional basic computer courses to improve the system's implementation in addition to the specific user training necessary for the success of DHIS2 in southwest Ethiopia.

### Limitations of the study and future research

Although we think our study will significantly aid future DHIS2 utilization in a limited resource setting, some limitations must be mentioned. This study did not include public health centers and private hospitals, and the findings were not supported by a qualitative study. Moreover, only self-reported questionnaires were used to collect the data for our study, which means there may be some response bias. Future studies should test the D&M model by adding net benefit, and the proposed model needs to be regularly tested, verified, and expanded in a variety of user and implementation contexts.

## Conclusion

The overall user satisfaction with using the district health information system in Ethiopia was low. The modified D&M model was found to be well-suited to evaluating the effectiveness of DHIS-2 in southwest Ethiopia. The SEM result showed that system quality, service quality, and computer literacy had a direct positive effect on system use and user satisfaction. In addition, system use and information quality had a direct positive effect on healthcare professionals “satisfaction with using DHIS 2. The relationship between system quality and user satisfaction with DHIS2 is mediated in part by system use. In addition, system use fully mediates the relationship between computer literacy and user satisfaction towards DHIS2. These findings assist implementers in understanding key areas for DHIS2 users. The most important factor for enhancing system use and healthcare professionals” satisfaction with DHIS2 was computer literacy. Accordingly, in addition to the specific user training required for the success of DHIS2 in Ethiopia, the manager should offer additional basic computer courses to the system users.

## Data Availability

The raw data supporting the conclusions of this article will be made available by the authors, without undue reservation.
