# Supplementary material for: Healthcare professionals' satisfaction toward the use of district health information system and its associated factors in southwest Ethiopia: using the information system success model
Source: Front Digit Health. 2023 Jul 17;5:1140933. doi: 10.3389/fdgth.2023.1140933 (PMC10389655; doi:10.3389/fdgth.2023.1140933)
Supplement: Supplementary file 1 [file Datasheet1.docx]

**Questionnaire**

1. **Questions related to socio-demographic factor**

| Se. No. | Questions | Responses |
| --- | --- | --- |
| 101 | Age in year | _____________ |
| 102 | Gender | 1. Female 2. Male |
| 103 | What is your Profession | 1. Doctors 2. Health officers 3. Nurse 4. Midwifery 5. Laboratory 6. Pharmacists 7. Radiologist 8. Physiotherapy 9. Optometry 10. HMIS staff 11. Health Data Entry 12. Management Secretaries 13. Information System Officer 14. Other, please specify_____________________ |
| 104 | Working Experience in Year | ___________________ |
| 105 | Monthly salary in Ethiopian birr | __________________ |

Please read the following statements concerning the DHIS2 satisfaction have in your hospital and, circle the number that best characterize your perception, **where 1 = Strongly Disagree, 2 = Disagree, 3 = Neutral, Agree, 4 = Agree, 5 = Strongly Agree**

1. **Questions related to user satisfaction**

| Se. No. | Statement | Strongly Disagree | Disagree | Neutral | Agree | Strongly Agree |
| --- | --- | --- | --- | --- | --- | --- |
| 201 | DHIS2 system help me to finish my work faster | 1 | 2 | 3 | 4 | 5 |
| 202 | DHIS2 system improves my productivity | 1 | 2 | 3 | 4 | 5 |
| 203 | I prefer the DHIS2 system than the paper record | 1 | 2 | 3 | 4 | 5 |
| 204 | The System has positive impact on quality of care | 1 | 2 | 3 | 4 | 5 |
| 205 | Overall, I am satisfied with the DHIS2 system | 1 | 2 | 3 | 4 | 5 |

1. **Questions related to system training**

| Se. No. | Variable | Possible answer | Skip |
| --- | --- | --- | --- |
| 301 | Have you taken digital health training(s)? | 1. Strongly Disagree 2. Disagree 3. Neutral 4. Agree 5. Strongly Agree |  |
| 302 | Have you taken Health Management Information System (HMIS) training(s)? | 1. Strongly Disagree 2. Disagree 3. Neutral 4. Agree 5. Strongly Agree |  |
| 304 | Have you taken DHIS2 training? | 1. Strongly Disagree 2. Disagree 3. Neutral 4. Agree 5. Strongly Agree | If your answer is 1 and 2, go to 305 |
| 305 | How many times you take | 1. One 2. Two 3. More than three |  |

1. **Questions related to computer literacy**

| No | Statement | Strongly Disagree | Disagree | Neutral | Agree | Strongly Agree |
| --- | --- | --- | --- | --- | --- | --- |
| 401 | I am interested in working with computers | 1 | 2 | 3 | 4 | 5 |
| 402 | I have moderate skill in using computers | 1 | 2 | 3 | 4 | 5 |
| 403 | I take computer trainings in the hospital | 1 | 2 | 3 | 4 | 5 |
| 404 | I feel that using computers will support me to be more efficient  in the future | 1 | 2 | 3 | 4 | 5 |

1. **Questions related to system quality**

| Se. No. | **Statement** | Strongly Disagree | Disagree | Neutral | Agree | Strongly Agree |
| --- | --- | --- | --- | --- | --- | --- |
| 501 | It is easy to learn | 1 | 2 | 3 | 4 | 5 |
| 502 | It is user friendly | 1 | 2 | 3 | 4 | 5 |
| 503 | Patient data transfer between department is easily | 1 | 2 | 3 | 4 | 5 |
| 504 | The system is stable | 1 | 2 | 3 | 4 | 5 |
| 505 | The system responds to my queries within an acceptable time. | 1 | 2 | 3 | 4 | 5 |
| 506 | I can retrieve the information I need easily | 1 | 2 | 3 | 4 | 5 |

1. **Questions related to information quality**

| Se. No. | Statement | Strongly Disagree | Disagree | Neutral | Agree | Strongly Agree |
| --- | --- | --- | --- | --- | --- | --- |
| 601 | The report is in a useful format | 1 | 2 | 3 | 4 | 5 |
| 602 | The information is clear | 1 | 2 | 3 | 4 | 5 |
| 603 | The information is complete | 1 | 2 | 3 | 4 | 5 |
| 604 | The information is up to date | 1 | 2 | 3 | 4 | 5 |
| 605 | The information is timely | 1 | 2 | 3 | 4 | 5 |
| 606 | The information is secured | 1 | 2 | 3 | 4 | 5 |
| 607 | The information is precise | 1 | 2 | 3 | 4 | 5 |

1. **Questions related to service quality**

| Se. No. | Statement | Strongly Disagree | Disagree | Neutral | Agree | Strongly Agree |
| --- | --- | --- | --- | --- | --- | --- |
| 701 | My supervisor is helpful | 1 | 2 | 3 | 4 | 5 |
| 702 | IT staff understand our need | 1 | 2 | 3 | 4 | 5 |
| 703 | The DHIS2 training is enough | 1 | 2 | 3 | 4 | 5 |
| 704 | I’m satisfied with user guideline | 1 | 2 | 3 | 4 | 5 |
| 705 | Computer access is enough | 1 | 2 | 3 | 4 | 5 |
| 706 | I can get technicians support easily | 1 | 2 | 3 | 4 | 5 |
| 707 | There is no frequently power interruption | 1 | 2 | 3 | 4 | 5 |
| 708 | There is back up power in my unit | 1 | 2 | 3 | 4 | 5 |
| 709 | Software problem get fixed in acceptable time frame | 1 | 2 | 3 | 4 | 5 |

1. **Questions related to usability of DHIS2 system**

| **No** | **Statement** | Strongly disagree | Disagree | neutral | Agree | Strongly agree |
| --- | --- | --- | --- | --- | --- | --- |
| 801 | I think that I would like to use the DHIS2 system frequently. | 1 | 2 | 3 | 4 | 5 |
| 802 | I found the system unnecessarily complex. | 1 | 2 | 3 | 4 | 5 |
| 803 | I thought the system is easy to use. | 1 | 2 | 3 | 4 | 5 |
| 804 | I think that I would need the support of a technical person to be able to use this system. | 1 | 2 | 3 | 4 | 5 |
| 805 | I found the various functions in this system were well integrated | 1 | 2 | 3 | 4 | 5 |
| 806 | I thought there was too much inconsistency in this system. | 1 | 2 | 3 | 4 | 5 |
| 807 | I would imagine that most people would learn to use DHIS2 system very quickly. | 1 | 2 | 3 | 4 | 5 |
| 808 | I found the system very cumbersome to use. | 1 | 2 | 3 | 4 | 5 |
| 809 | I felt very confident using the DHIS2 system. | 1 | 2 | 3 | 4 | 5 |
| 810 | I needed to learn a lot of things before I could get going with this system. | 1 | 2 | 3 | 4 | 5 |

1. **Questions related to attitude to use DHIS2 system**

| **No** | **Statement** | Strongly disagree | Disagree | neutral | Agree | Strongly agree |
| --- | --- | --- | --- | --- | --- | --- |
| 901 | Using district health information system (DHIS-2) is good idea | 1 | 2 | 3 | 4 | 5 |
| 902 | District health information system (DHIS-2) makes work more interesting | 1 | 2 | 3 | 4 | 5 |
| 903 | I like work with district health information system (DHIS-2) | 1 | 2 | 3 | 4 | 5 |
| 904 | I have positive feeling toward the use of district health information system (DHIS-2) | 1 | 2 | 3 | 4 | 5 |
